# Prevalence of and factors associated with diarrhoeal diseases among children under five in Malaysia: a cross-sectional study 2016

**DOI:** 10.1186/s12889-018-6266-z

**Published:** 2018-12-11

**Authors:** Fazly Azry Abdul Aziz, Noor Ani Ahmad, Mohamad Aznuddin Abdul Razak, Maisarah Omar, Noraida Mohamad Kasim, Muslimah Yusof, Rajini Sooryanarayana, Rasidah Jamaludin, Chan Ying Ying

**Affiliations:** Institute for Public Health, Ministry of Health Malaysia, Jalan Bangsar, 50590 Kuala Lumpur, Malaysia

## Abstract

**Background:**

Globally, diarrhoea is one of the major causes of morbidity and mortality among children under than 5 years of age. There is a scarcity of published data on acute gastroenteritis (AGE) prevalence in Malaysia among children. This study aims to determine factors associated with diarrhoea in children aged less than 5 years in Malaysia.

**Method:**

Data from the National Health and Morbidity Survey 2016 conducted by Ministry of Health was analysed. This nationwide survey involved 15,188 children below five years old. The survey was carried out using a two-stage stratified sampling design to ensure national representativeness. The Questionnaire from UNICEF’s Multiple Indicator Cluster Survey (MCIS) was adapted to suit local requirements. Analysis was done using SPSS Version 23. Descriptive followed by multiple logistic regression were done to identify relevant factors.

**Result:**

The prevalence of diarrhoea among children under five in Malaysia was 4.4% (95% CI: 3.8,5.2). Analysis using logistic regression indicated that only ethnicity and usage of untreated water were significantly associated with diarrhoea among children after controlling for relevant factors. By ethnicity, children in the ‘Other Bumiputera’ group had 2.5 times the odds of having diarrhoea compared to children of Malay ethnicity. Children of Indian ethnicity were also at higher risk, at almost double the odds, as well as other ethnic groups (1.5 times). Children who used untreated water supply were two times more likely to develop diarrhoea.

**Conclusion:**

There is a higher risk of diarrhoea among children of ‘Other Bumiputera’ ethnicity, Indian ethnicities, and other ethnic groups and those who consume untreated water. Strategies to reduce diarrhoea among children should be targeted towards these at-risk populations. In addition, the Government must strive to ensure universal access to treated clean water in Malaysia and the Ministry of Health must focus on raising awareness on how to prevent diarrhoea.

## Background

Diarrhoeal disease is among the leading causes of mortality and morbidity among children under 5 years. WHO estimates that, worldwide, 525,000 children under five years die due to diarrhoeal diseases each year with 1.7 billion cases of diarrhoeal disease every year [1]. In developing countries, children under three years’ experience on average three episodes of diarrhoea every year. Each episode contributes to significant nutrition deprivation which is necessary for child growth [2].

The prevalence of diarrhoea in young children is linked to socioeconomic status. In North Sudan, a study found that 28% of children below five years had diarrhoea [3]. Another study done in Eastern Ethiopia revealed a diarrhoea prevalence of 21.5% [4]. Both these countries fall under the low-income group according to the World Bank [5]. In middle income countries, the prevalence is lower, a study done in 2016 for Thailand reported a prevalence of 4.9% while Vietnam reported 8.6% in 2014 [6, 7].

Based on the 2006 National Health and Morbidity Survey (NHMS), the overall prevalence of diarrhoea in children below 5 years old in Malaysia was 4.5% [8]. Detailed analysis of the NHMS done in year 2011 found that children below 5 years old were 33% higher compared to 5–9 years old aged group [9]. Although Malaysia enjoys a high coverage of treated water, diarrhoea episodes among children are still reported. We found a scarcity of published data on acute gastroenteritis (AGE) prevalence in Malaysia among children. Information on the risk factors of diarrhoea in children will benefit intervention strategies to halt this problem. As such, this study aims to determine factors associated with diarrhoea in children aged less than 5 years in Malaysia.

## Methods

### Sampling

This study used data from Malaysia’s National Health and Morbidity Survey (NHMS) 2016. The NHMS is a series of nationwide surveys conducted on a regular basis to support the Ministry of Health in monitoring and evaluating the health of the Nation. This survey was designed to capture a nationally representative sample of residents in non-Institutional living quarters in both urban and rural localities in all 13 states and 2 Federal Territories in Malaysia. The sample of children under five was selected based on a sampling frame of live births provided by the National Registration Department (NRD). Details of the Survey methodology was also mention in another published article [10].

As NHMS covered broad topics under Maternal and Child Health which includes immunization as the main topic and diarrhoea as one of sub topic. Thus, the sample size was calculated based on an 50% estimated prevalence of immunization and design effect of 1.5. The final sample size was inflated to 17,330, which were then allocated to the states in equal proportions to compensate for a 50% expected non-response rate [11].

The Questionnaire for this study was based on the WHO Multiple Indicator Cluster Survey (MICS), adapted with permission to suit Malaysian requirements [13]. Permission for its use was authorised by UNICEF. This questionnaire was administered in two languages and anonymous. Data collection started on 17 February 2016 in Sabah and Sarawak, and on 25 February 2016 in Peninsular Malaysia and was completed on 26 May 2016.

Bilingual questionnaire; English and Bahasa Malaysia languages were used for this survey. Questionnaire in Bahasa Malaysia version was translated from English by content experts with English proficiency then back-translated into English by English speaking experts with Bahasa Malaysia proficiency. A pilot test was done using the Bahasa Malaysia version involving 30 individuals from Malaysia’s major ethnicities; Malays, Chinese and Indians, and from both low and highly educated individuals. Cognitive testing which looks into understanding of the content and terms used was found to be satisfactory. The questionnaire was conducted by face-to-face interview using mobile devices, with the parent or caregiver of children aged 0–59 months who responded to this module.

### Variable definitions

In this study, diarrhoea was defined by a question “In the last 2 weeks, has …… (child’s name) had diarrhoea” on the passage of three or more loose/watery stools per day with/without blood in the stools in the past 2 weeks prior to data collection, as perceived by the mother/caregiver. The response options were either ‘YES’ or ‘NO’. Water sources were also inquired into by the question “What is the main source of drinking water for this household?”, and for analysis it was further categorised into - either Treated (Boil, add bleach/chlorine, use water filter) or Untreated water (others or nothing has been done). Method of garbage disposal was categorised into groups of sanitary or unsanitary waste disposal. Sanitary waste disposal included those collected by a local authority/management regularly, collected by local authority/management irregularly and buried outside the house. Unsanitary waste disposal consisted of open burning, throw into the drain, river or sea or anywhere. For latrines, the options were regrouped into either sanitary latrine (child used toilet, put/rinsed into the toilet) or unsanitary latrines (thrown into drain, ditch, river or sea, thrown in garbage, left open and others).

Locality was classified into Urban and Rural based on Department of Statistics Malaysia definitions: Urban area was classified as an area with a population above 10,000 and at least 60% of its population aged above 15 years involved in non-agricultural activities. Rural area was classified for areas that did not meet those criteria.

In this study, five major ethnic groups were identified. Three main ethnicities include Malay, Chinese and Indian descent with another two comprising ‘Other Bumiputera’ (local ethnic groups of the East Malaysian states of Sabah and Sarawak) and ‘Others’ (foreigners, immigrants, both legal and illegal, residing in Malaysia). For children, their ethnicity was based on paternal ethnicity. Citizenship was categorised as either Malaysian citizens or non-Malaysian.

The Malaysian education system was used to further categorise both parents’ education level. No formal education means the respondent had not attended any formal schooling while primary education applied to those who completed up to six years of primary school. Secondary education applied to those who finished 11 years of formal schooling and, finally, respondents with a minimum qualification of a diploma and/or higher were coded as having tertiary education.

Occupation of parents was clustered into either working in public sector, private sector, self-employed or housewife/unemployed. The pooled income of family members was further divided into quantiles to categorise household income with the first quintile representing the lowest 20% and the fifth quintile representing the highest 20% of income.

The survey or final weight was calculated based on design weight and non-response weight and plan for analysis was created. To ensure that the data can be inferred to the Malaysian population, particularly for ethnicity, post stratification adjustment was made.

To obtain the association of diarrhoea among children with factors such as sociodemographic profile, treated water, waste and latrine disposal, we used multiple logistic regression. The adjusted odd ratio (aOR) of having diarrhoea, with 95% confidence intervals (CIs), and *P* value < 0.05 were used to describe associations.

First, we did univariate analysis to determine the associations between diarrhoea and other associated factors using chi square and binary logistic regression. Those with a *P* value less than 0.05 were included in the final multivariable model. Taking into consideration the complexity of the sampling design, analysis was done using complex sample module in the IBM Statistical Package of Social Sciences (SPSS) for Windows version 23 (IBM Corp., Armonk, NY, USA),.

## Results

### Respondents profile

From a total of 16,966 eligible children, 15,188 responded to this survey giving a response rate of 89.5%. Table 1 illustrates the profile of respondents’ children under five years old. Most of the respondents resided in urban areas. Majority of the children were Malays and more than half of the parents have up to secondary level education.

**Table 1:** Sociodemographic Profile of Respondent

	*n*	Percentage (%)	95% CI	
			Lower	Upper
Strata				
Urban	8997	64.7	63.4	65.9
Rural	6191	35.3	34.1	36.6
Ethnicity				
Malay	10,686	62.3	60.6	64
Chinese	1737	15.9	14.4	17.5
Indian	642	4.9	4.1	5.8
Others Bumiputera	1789	14.7	13.9	15.7
Others	294	2.2	1.9	2.7
Fathers' Education				
No formal education/Primary	1993	14.2	13.1	15.3
Secondary	7812	54.1	52.3	56
Higher	4597	31.7	29.9	33.6
Mothers Education				
No formal education/Primary	1834	12.5	11.5	13.5
Secondary	7720	51.8	50.0	53.6
Higher	5472	35.7	33.9	37.6
Total Household Income				
Poorest	3088	19.6	18.4	20.8
Second	2998	19.3	18	20.6
Middle	3096	20.4	19	21.8
Fourth	3031	18.8	17.4	20.3
Richest	2885	21.9	20.3	23.6
Mothers' Occupation				
Public Sector	3755	19.70	18.4	21.1
Private Sector	3238	25.1	23.4	26.9
Self Employed	1108	7.1	6.2	8.2
Housewife/Unemployed/Student	6938	48.1	46.3	49.8
Fathers' Occupation				
Public sector	3575	19.7	18.3	21.2
Private sector	6772	51.4	49.5	53.2
Self-employed	3954	28.0	26.4	29.6
Unemployed/student	136	0.9	0.7	1.2
Treated Water				
Treated Water	14,914	97.9	97.5	98.3
Untreated Water	272	2.1	1.7	2.5
Waste Disposal				
Sanitary Waste	11,924	78.6	77.3	79.8
Unsanitary	3160	20.5	19.3	21.8
Latrines				
Sanitary	14,768	96.8	96.3	97.2
Unsanitary	412	3.2	2.8	3.7

With regards to parental occupation, almost half of the mothers were housewives/ unemployed/ students while more than half of the fathers work in the public sector. From a hygiene point of view, a majority of household have access to treated water, sanitary latrines and proper waste disposal in their homes (Table 1).

### Prevalence of diarrhoea and associated factors among children

The prevalence of diarrhoea among children under five in Malaysia from this study was 4.4% (95% CI = 3.8–5.2). There was no difference between boys and girls. Overlooking nationality, children of permanent residents registered the highest prevalence of diarrhoea at 6.6% (95% CI = 1.3–27.2). By ethnicity, ‘Other Bumiputeras’, i.e., children from Sabah and Sarawak, recorded the highest prevalence, 8.8% (95% CI = 6.9–11.3). By parents’ formal education, diarrhoea most commonly occurred among children whose father has no formal /primary level education, 5.0% (95% CI = 3.5–7.0) and, even higher, among those whose mother has no formal education, 7% (95 CI = 4.5–10.9). In terms of household income quintile, the lowest household income group recorded the highest prevalence of childhood diarrhoea (5.8%; (95% CI = 4.4–7.6) compared to 3.2% e (95% CI = 1.9–5.3) in the highest income quintile.

The study revealed that for father’s occupation, the unemployed group registered the highest prevalence of 5.5% (95% CI = 2.0–14.3) while for mothers, highest prevalence was among those working in the private sector, 4.9% (95% CI = 3.3–7.3). Having Untreated water showed a significantly much higher prevalence of childhood diarrhoea, 13.4% (95% CI = 7.9–21.8) compared to having treated water. Diarrhoea in under-fives was also more common among those with Unsanitary waste disposal, 6.8% (95% CI = 5.3–8.7) as well as those with unsanitary latrines, 6.3% (95% CI = 4.0–9.6).

Multiple logistic regression revealed only factors significantly associated to diarrhoea among children; ethnicity and untreated water supply. Specifically, compared to the Malays, the odds of having diarrhoea among “other Bumiputras” children were 2.4 followed by Indian children (1.7) after controlling for other factors. Families using untreated water faced twice higher odds of having diarrhoea compared with families using treated water after controlling for other factors. (Table 2).

**Table 2:** Associated Factor of diarrhoea via multiple logistic regression

Variables	Crude				Adjusted odds ratio, aOR		
	*n*	Odds ratio, OR	95% CI	*P* Value	aOR	95% CI	*P* Value
Strata							
Urban	8987	1.249	1.08,1.44	0.002			NS
Rural	6177	1					
Ethnicity							
Malay	10,665	1			1		
Chinese	1737	1.05	0.82,1.34	0.696	1.03	0.80,1.33	> 0.05
Indian	641	1.86	1.38,2.53	< 0.001	1.70	**1.22,2.36**	**0.002**
Others Bumiputera	1788	2.64	2.21,3.14	< 0.001	2.40	**1.97,2.93**	**< 0.001**
Others	293	1.53	0.95,2.46	0.08	1.57	0.95,2.60	0.08
Father's Education level							
No Formal Education/Primary	1990	1.61	1.28,2.02	< 0.001			NS
Secondary	7799	1.30	1.09,1.55	0.003			
Tertiary	4589	1					
Mother’s Occupation							
Public Sector	3751	1					
Private Sector	3233	0.99	0.80,1.24	0.985			
Self Employed	1103	0.78	0.55,1.10	0.145			
Unemployed/Housewife/Student	6928	1.20	1.00,1.43	0.049			
Fathers Occupation							
Public Sector	3567	1					
Private Sector	6762	1.13	0.94,1.37	0.199			
Self Employed	3948	1.15	0.93,1.42	0.190			
Unemployed/Student	136	1.44	0.72,2.89	0.301			
Mothers Education							
No formal education/Primary	1883	1.84	1.48,2.28	< 0.001			NS
Secondary	7708	1.29	1.09,1.51	0.003			
Higher	5461	1					
Total Household Income							
Poorest	3081	1.33	1.06,1.66	0.016			NS
Second	2993	1.11	0.88,1.41	0.381			
Middle	3090	1.24	0.98,1.56	0.07			
Fourth	3029	1.01	0.79,1.28	0.946			
Richest	2881	1					
Water Supply							
Treated	14,890	1			**1**		
Untreated	272	3.39	2.42,4.74	< 0.001	**2.04**	**1.40,2.97**	**< 0.001**
Waste Disposal							
Sanitary Waste Disposal	11,906	1					
Unsanitary Waste Disposal	3155	1.46	1.24,1.71	< 0.001			NS
Others	99	1.72	0.82,3.57	0.143			
Sanitary and Unsanitary							
Sanitary Latrines	14,744	1					
Unsanitary Latrines	412	2.14	1.55,2.96	< 0.001			NS

*NS not significant

*OR* odd ratio

*AoR* adjusted odd ratio

*CI* confidence interval

Significant results are bold

## Discussion

Numerous factors associated with increased risk in diarrhoea among children under five have been identified. In the present study, we found significant differences in diarrhoea prevalence by ethnicity, parents’ education and occupation, household income, water supply, sanitation and waste disposal. The differences were all expected based on what is known regarding the risk factors for diarrhoeal diseases. Our findings correlate with a study in India which reported that prevalence of diarrhoea was positively associated with occupation and education of mother, source of water and toilet facility [11]. The same study also identified storage of water, lack of hand washing, feeding habits, immunization and rural residence, factors which were not included in our study [12]. Meanwhile, a study done in Baghdad showed that children were more at risk when the mother is unemployed and has lower education, and mother and father have poor nutritional knowledge [14].

The present overall prevalence of diarrhoea among children under five was 4.4%. In NHMS 2006, prevalence of diarrhoea was reported at 4.5% [8]. Although the rate is not high, our study demonstrates that diarrhoea prevalence among children less than five years has not shown any improvement in the past 10 years. Although Malaysia has rapidly improved its health and sanitation facilities, and there is now wider access to clean water and proper education, this condition may be contributed by the influx of immigrants to our country recently as NHMS targeted all residents in Malaysia irrespective of the nationality status. For comparison, a study done in 2013 using the same MICS survey tool in Bangladesh and Mongolia reported that prevalence of diarrhoea among children under five was 3.9 and 8.2%, respectively [14].

Highest prevalence was seen in Other Bumiputeras (8.8%), Indians (5.3%), others (5%) and the least was among Chinese children (3.3%). The previous study done (NHMS 2006) showed the Other Bumiputeras also recorded the highest level of prevalence of diarrhoea of 5.8% compared to other major ethnic groups, with the Chinese least likely to report acute diarrhoea. The reasons for such differences are not well understood, however the finding could be attributed to genetic or sociocultural differences between the ethnicities, which may be related to dietary and culinary practices or risk of acquiring gastrointestinal infections [9].

Prevalence of diarrhoea in Malaysia using untreated water was 13.4%. This is clearly supported by a study done in neighbouring Indonesia where they found a significant association between children with diarrhoea and unsafe drinking water source [15]. Meanwhile, a study done in Ibadan, Nigeria showed an increased risk of diarrhoea among children whose caregivers/mothers collected drinking water from a storage container by dipping into it with any other container [16]. Contaminated water may carry diarrhoea causing pathogens that may lead diarrhoea.

By education level, children of parents with lower education levels were more prone to have diarrhoea compared to parents with tertiary education levels. A similar study in India found a 13% lower risk of diarrhoea among educated mothers versus uneducated mothers [12]. Another study done in Ghana, stated that the prevalence of diarrhoea varied according to education of mother, being significantly lower among children with educated mothers (secondary and higher) than among children of mothers with no or primary level education [17]. In Malta, the majority of cases were found to have lower education [18]. Education provides knowledge of hygiene, good feeding and weaning practises, early recognition of symptoms and, thus, timeliness in responding to childhood illnesses [19]. Be that as it may, with regards to diarrhoea prevention, parents and caregivers can be informed and trained on hygienic practices and disease control regardless of their low education level. This is the aim and function of public health strategies – to develop programmes suited to the capabilities of the target communities.

Our survey observed that the poorest with prevalence of 5.8% were the most vulnerable to diarrhoea among the various household income groups. A study done in Thailand showed identical results with a diarrhoea prevalence rate of 9.8% [20]. A study conducted in Malawi mentioned that the father’s job or poor income of a head of the household is also a risk factor for diarrhoea in children [21]. In Bangladesh, a study showed the effect of maternal education and family income on diarrhoea, where they found high family income was associated with more than 40% of risk reduction [22]. This could be explained by children living in poorer households having higher diarrhoea rates than their wealthier counterparts due to sanitary facilities, unsanitary environments in the home and poor child hygiene.

Our study concluded that children from homes with unsanitary latrines and/or waste disposal were more likely to have diarrhoea compared to those with sanitary latrines and/or waste disposal. This is not surprising. Prevalence of diarrhoea for unsanitary waste disposal was 6.8% while for unsanitary latrines it stood at 6.3%. A recent study done in Banten, Indonesia, discovered that mothers who defecated at latrines have fewer children with diarrhoea than mothers who defecated at other places [15]. Improved access to sanitation reduces the risk of contracting diarrhoea by 2.2 percentage points, as shown by a study done in India [23]. An earlier study done in Burkina Fasso, found that children whose mothers regularly disposed of feces in the latrines had an approximate 40% reduction in diarrhoea rate [24]. In India, another study showed that, children from households in which the toilet facilities were not available within the house were more likely to develop diarrhoea compared with children from households with toilet facilities [12]. The presence of latrines increases the chance of safe disposal of faeces, reduces the risk of contact between the causative organisms of diarrhoea and the host. Similarly, with regards to proper waste disposal, unsafe refuse disposal showed a statistically significant relationship with childhood diarrhoea in Atwima Nwabiagya District in Ghana. Children who lived in households where unsafe refuse disposal was practised were two times more likely to suffer diarrhoea [25]. This result was consistent with studies conducted In Ethiopia which also found that improper refuse disposal was associated with a higher risk of childhood diarrhoea [26].

Finally, after controlling for various factors, multivariate analysis showed that only the associations with ethnicity and untreated water remained significantly associated with diarrhoea among children under five years old.

## Strengths and limitation

The strength of this study is the analysis based on a large nationally representative sample that allowed for a reliable inference to Malaysian children within a similar age-group. This survey used robust standard WHO MICS questionnaire, which is advantageous for international comparison.

Causal relationship between identified factors and diarrhoea was unable to be identified due to cross-sectional nature of this study. In addition, we only did face and content validity of the questionnaire used in this study.

Diarrhoea prevalence was based on self-reported screening and was not further confirmed.

## Conclusion

Diarrhoea among children is a very important issue that needs to be addressed. From this study, we have found that diarrhoea in Malaysia was higher in Indian and Other Bumiputeras by ethnicity and usage of untreated water. Thus, healthcare strategy and campaign should be focused on specific ethnicity and universal treated water must be available among all residents of Malaysia.

## Abbreviations

NHMS: National Health and Morbidity Survey
UNICEF MCIS: United Nation Children’s Fund Multiple Indicator Cluster Survey
WHO: World Health Organisation
